# Chronic Cervical Catarrh of the Uterus

**Published:** 1900-11-03

**Authors:** 


### Hospital Clinics and Medical Progress.

### CHRONIC CERVICAL CATARRH OF THE UTERUS.—*Continued.*

The pathology of the condition is fairly simple and need not detain us. The leucorrhœa is caused by increase and enlargement of the mucous glands of the cervix, and the extension of the glandular surface is seen in the erosions or catarrhal patches on its vaginal surface. These are no longer considered ulcerations; and though we see many patients with a history of ulceration of the womb, I do not think this is due so much either to the frequency of true ulceration, or to mistakes in diagnosis, as to patients' desire for a diagnosis, and the practitioner's wish to satisfy this, and to give them something they can understand, so that they may submit patiently to treatment. That these erosions do resemble ulcers in their appearance and that they easily bleed, is certain, but there is no loss of substance as in a true ulcer; on the contrary, there is an increase, development, and extension of normal structures. The raw appearance is due to the vascular underlying tissue seen through the single layer of small columnar cells which replace the normal shining pavement epithelium of the vaginal surface of the cervix, and the foldings of the glandular recesses give the characteristic granular appearance. If these recesses and ducts become choked or blocked, and distended with secretion, cysts are produced, and they may grow and burst on the surface, producing follicular erosion. True ulceration may be present if the superficial epithelium of the cervix be abraded and the papillæ on its vaginal surface destroyed in definite areas. Lacerations of the cervix result in catarrh, as they cause many other pathological conditions, and this of a severe and persistent type. They permit the inroads of bacteria into damaged tissues, they evert and expose the cervical mucosa to constant irritation, and are probably the most frequent cause of cervical catarrh in multiparæ.

*The treatment* of chronic cervical catarrh is, as we have seen, both constitutional and local. The former is conducted on general principles. The patient's habits and environment are carefully enquired into and faulty hygienic conditions remedied. Regular hours, plain nourishing food taken at proper intervals, and a fair amount of outdoor exercise, will be very helpful. It is necessary to obtain a clear idea of the patient's habits, diet, and surroundings, and not to be put off with generalities of description. What the public regard as normal and proper does not necessarily meet with the approval of the medical attendant, and it is not at all unlikely that precise enquiry on these points will clear up the etiology of the case, and offer useful hints for treatment. Nervous symptoms of a subjective character will benefit by rest and change of air, and this will often secure the sexual rest that is very desirable in these cases. The nervous tone of the patient will greatly improve, and her irritability, depression, or capricious temper disappear. As a general tonic I find iron and arsenic the most valuable in this disease. A pill containing 2 gr. of the dried sulphate of iron, $\frac{1}{40}$ to $\frac{1}{20}$ of a grain of arsenious acid, $\frac{1}{4}$ gr. extract of nux vomica, taken after meals three times a day, will be found serviceable.

The nux vomica may be replaced by $\frac{1}{40}$ gr. strychnine, or quinine, and a small dose of aloin (gr. $\frac{1}{20}$ to $\frac{1}{5}$) added to prevent constipation, where this is troublesome. Dyspeptic symptoms may be met with appropriate remedies and a strictly defined dietary. And this should ensure that the woman gets enough to eat, including a fair proportion of red meat. In the lower, and even amongst the middle, classes, it is not always that patients are sustained by a proper, nourishing diet. Dinner, e.g., is a neglected meal. Perhaps the husband dines in town, and his wife will not incur the expense of a suitable dinner, or, if it falls upon herself, the trouble of cooking it. In these cases, if we include eggs, fish, or a chop with breakfast or tea, we are more likely to ensure sufficient nourishment than by depending on a scamped dinner for the principal animal food of the day. To make a woman eat well and digest well is the secret of cure in many conditions of breakdown, associated or not with affections of the genital tract. Where there is some dyspepsia and constipation we may replace by, or combine with the above iron pill a small dose of rhubarb as a stomachic.

So much for drugs, diet, and general hygiene, all of which must be prescribed with a watchful eye on diathesis. And so much can be obtained from such treatment on general principles, that in young, unmarried, and neurotic women it is justifiable in slight cases to give it a trial before resorting to local treatment. Of course, local treatment is usually necessary, but it is not always good to direct a woman's attention to her pelvic organs. It is apt to fix itself there, and in neurotic women this is very undesirable. But where the disease does not speedily improve local treatment cannot be avoided. I imagine that the great faults in the local treatment of chronic cervical catarrh consist in treating it too vigorously at first and by not sufficiently radical methods in advanced cases. To ceaselessly cauterise erosions and attack the cervical mucosa with powerful astringents on the appearance of a mild catarrh is as absurd as it would be to apply the same methods to the nasal mucous membrane. A judicious treatment begun early and carried out patiently will generally cure the disease within a reasonable time.

The vaginal douche given in the recumbent or semi-recumbent position once or twice daily will be found valuable. It need not be very hot, unless the patient have pain or hæmorrhage (and therefore something more than cervical catarrh); but it should be copious enough to be cleansing, and will be better for being antiseptic. Two to four pints of hot water, containing a teaspoonful to the pint of bicarbonate of soda, will serve to commence with, or a quart of plain hot water followed by a pint in which half an ounce of boracic acid or boroglyceride has been dissolved. This should be given at least once a day. Some surgeons use with success more astringent applications: sulphate of zinc, a dram to the pint douche, to promote the replacement of normal squamous epithelium over the erosions on the vaginal surface of the cervix: sulphate of alum or copper, ʒij. to the pint, are also recommended and may be used if the mild antiseptic douche fail, such as the boracic acid or 1–3000 mercuric iodide.

(*To be continued.*)

